# Mechanism of DNA Interaction and Translocation by the Replicase of a Circular Rep-Encoding Single-Stranded DNA Virus

**DOI:** 10.1128/mBio.00763-21

**Published:** 2021-07-27

**Authors:** Elvira Tarasova, Sonali Dhindwal, Matthew Popp, Sakeenah Hussain, Reza Khayat

**Affiliations:** a Department of Chemistry and Biochemistry, The City College of New York, New York, New York, USA; b Graduate Program in Biochemistry, The Graduate Center of the City University of New York, New York, New York, USA; c Department of Chemistry, Queens College, New York, New York, USA; UCLA, CNSI/MIMG; University of North Carolina, Chapel Hill

**Keywords:** CRESS-DNA, replicase, rolling circle replication, cryo-EM, helicase

## Abstract

Circular Rep-encoding single-stranded DNA (CRESS-DNA) viruses infect members from all three domains of life (*Archaea*, *Prokarya*, and *Eukarya*). The replicase (Rep) from these viruses is responsible for initiating rolling circle replication (RCR) of their genomes. Rep is a multifunctional enzyme responsible for nicking and ligating ssDNA and unwinding double-stranded DNA (dsDNA). We report the structure of porcine circovirus 2 (PCV2) Rep bound to ADP and single-stranded DNA (ssDNA), and Rep bound to ADP and double-stranded DNA (dsDNA). The structures demonstrate Rep to be a member of the superfamily 3 (SF3) of ATPases Associated with diverse cellular Activities (AAA**+**) superfamily clade 4. At the Rep N terminus is an endonuclease domain (*ED*) that is responsible for ssDNA nicking and ligation, in the center of Rep is an oligomerization domain (*OD*) responsible for hexamerization, and at the C terminus is an ATPase domain (*AD*) responsible for ssDNA/dsDNA interaction and translocation. The Rep *AD* binds to DNA such that the *ED* faces the replication fork. The six *AD* spiral around the DNA to interact with the backbone phosphates from four consecutive nucleotides. Three of the six *AD* are able to sense the backbone phosphates from the second strand of dsDNA. Heterogeneous classification of the data demonstrates the *ED* and *AD* to be mobile. Furthermore, we demonstrate that Rep exhibits basal nucleoside triphosphatase (NTPase) activity.

## INTRODUCTION

Circular replicase (Rep)-encoding single-stranded DNA (CRESS-DNA) viruses are a diverse and widely prevalent group of viruses that infect *Archaea*, *Eubacteria*, and *Eukarya* domains of life (1–6). CRESS-DNA viral genomes vary from 1 kilonucleotide (knt) to 25 knt (7, 8). Rep is responsible for initiating, progressing, and completing rolling circle replication (RCR) of the genome. RCR was described more than a half century ago by Gilbert and Dressler as the mechanism of genome replication by the bacteriophage ϕX174 (1). RCR is ubiquitously used by nature for the replication of phage/viral DNA, bacterial plasmids, and eukaryotic helitrons (transposons) (2, 9–17). While a conceptual mechanism of RCR is well documented, its structural mechanism is desperately lacking (4). CRESS-DNA virus RCR proceeds after conversion of the ssDNA to double-stranded DNA (dsDNA). Rep binds to a sequence-specific origin of replication (*ori*) to unwind the genome and generate a cruciform structure—a process that is possibly accompanied with ATP hydrolysis by Rep. Rep nicks the (+) strand of the cruciform at a sequence-specific site to generate a free 3′-OH end for leading-strand DNA synthesis, and itself becomes covalently attached to the 5′-PO_4_ of the (+) strand. Replication continues to generate a complete (+) strand with several nucleotides beyond the start site. A second round of cleavage by Rep liberates the 3′-OH of the same strand. Rep then ligates the ssDNA 5′-PO_4_ attached to itself to the recently generated 3′-OH of the same strand to generate a circular ssDNA (4). DNA polymerization is accomplished by either cellular or viral/phage-encoded DNA polymerase. DNA strand separation is accomplished by either cellular or viral/phage helicases (Rep).

The nuclear magnetic resonance (NMR) and crystal structure of an N-terminal fragment of Rep identify this domain to be an HUH (His-hydrophobic-His motif) endonuclease domain (*ED*) (18, 19). *ED* is responsible for the nicking and ligation functions described above. The sequence and structural similarity shared between the Rep *ED*, *ED* from relaxases (enzymes responsible for RCR of bacterial plasmids), and *ED* from transposases suggests that these domains are evolutionarily related (6, 18, 20–22). Here, we describe the structure of a Rep from a circovirus, a CRESS-DNA virus, that encompasses the region C terminal to the *ED*. The *Circoviridae* family of viruses are members of the *Cressdnaviricota* phylum. This family is categorized into the *Cyclovirus* and *Circovirus* genera (23). Genetic material associated with the *Cyclovirus* genus has been identified from both vertebrates and invertebrates; however, a definitive host remains to be identified (24). Members of the *Circovirus* genus are widely distributed in nature and have been documented to infect terrestrial, aquatic, and avian animals (24, 25). Porcine circovirus 2 (PCV2) is the prototypical representative of the *Circovirus* genus as it has received the greatest attention due to its detrimental effect on the swine industry (26–28). To understand the architecture of Reps from CRESS-DNA viruses and how they interact with dsDNA and ssDNA to unwind dsDNA, we determined the structure of PCV2b Rep bound to ssDNA and dsDNA using cryo-electron microscopy (cryo-EM) to 3.8-Å and 4.4-Å resolution, respectively. The cryo-EM structures demonstrate Rep to belong to the superfamily 3 (SF3) of the ATPases Associated with diverse cellular Activities (AAA**+**) superfamily (clade 4). The structures further demonstrate the *ED* to be mobile, Rep to form a hexamer with ss/dsDNA bound to its central channel, Rep to use an oligomerization domain (*OD*) for hexamerization, and the Rep’s ATPase domains (*AD*) to adopt a spiral staircase arrangement around ssDNA/dsDNA and demonstrate a sequential mode of ATP hydrolysis and direction of ssDNA translocation. We further demonstrate that Rep exhibits basal nucleoside triphosphatase (NTPase) activity.

## RESULTS

### Cryo-EM structure of PCV2 Rep is a hexamer with a mobile domain.

**FIG 1:**
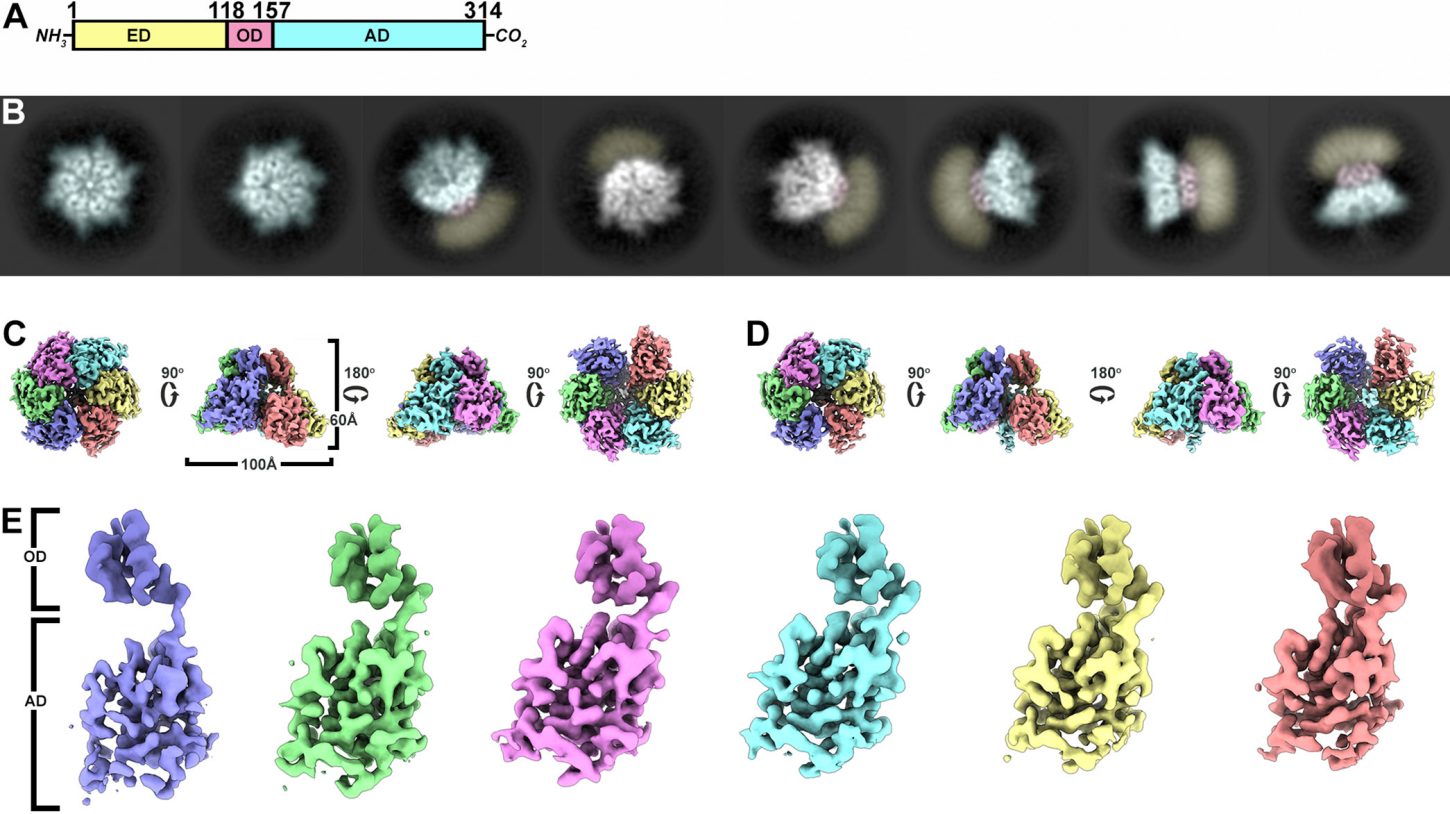
Architecture of PCV2 Rep-DNA. (A) Schematic of Rep with the endonuclease domain (ED, pastel yellow), oligomerization domain (OD, pastel red), and ATPase domain (AD, pastel cyan) labeled. The domain boundaries are identified by the amino acid numbers written at the top. (B) 2D class averages demonstrating different orientations of Rep visualized during cryo-EM imaging. The averages have been computationally color coded to identify the *ED*, *OD*, and *AD* using the color convention described above. (C) Orthogonal views of the Rep-ssDNA cryo-EM map showing top, side, and bottom views. The six subunits are color coded consistently throughout this paper. The *OD* is colored slightly darker than the *AD*. (D) Orthogonal views of the Rep-dsDNA cryo-EM map with orientations comparable to panel C. (E) Segmented subunits of the Rep-ssDNA cryo-EM map. The *OD* and *AD* are labeled on the left side. The *OD* of the six subunits were superposed to highlight the rigid body movement of the *AD*. Images in panels C to E generated using UCSF Chimera X.

Cryo-EM two-dimensional (2D) class averages identify top, tilted, and side views of Rep in ice (Fig. 1B). Top and tilted views demonstrate that Rep is a hexamer. The side views demonstrate that Rep can be described by three domains: (i) a featureless and presumably mobile domain, (ii) a featureful small domain that appears to form a collar-like structure, and (iii) a larger featureful domain neighboring the collar-like domain (Fig. 1B). While some side view classes provide low-resolution information on the first-described domain, other side view classes do not identify any features for this domain (Fig. 1B). Given that SDS-PAGE analysis identifies a single band for the Rep protein, the absence of signal for this domain is indicative of a domain that adopts distinct positions in space (i.e., a mobile domain).

### PCV2 Rep is an SF3 helicase.

**FIG 2:**
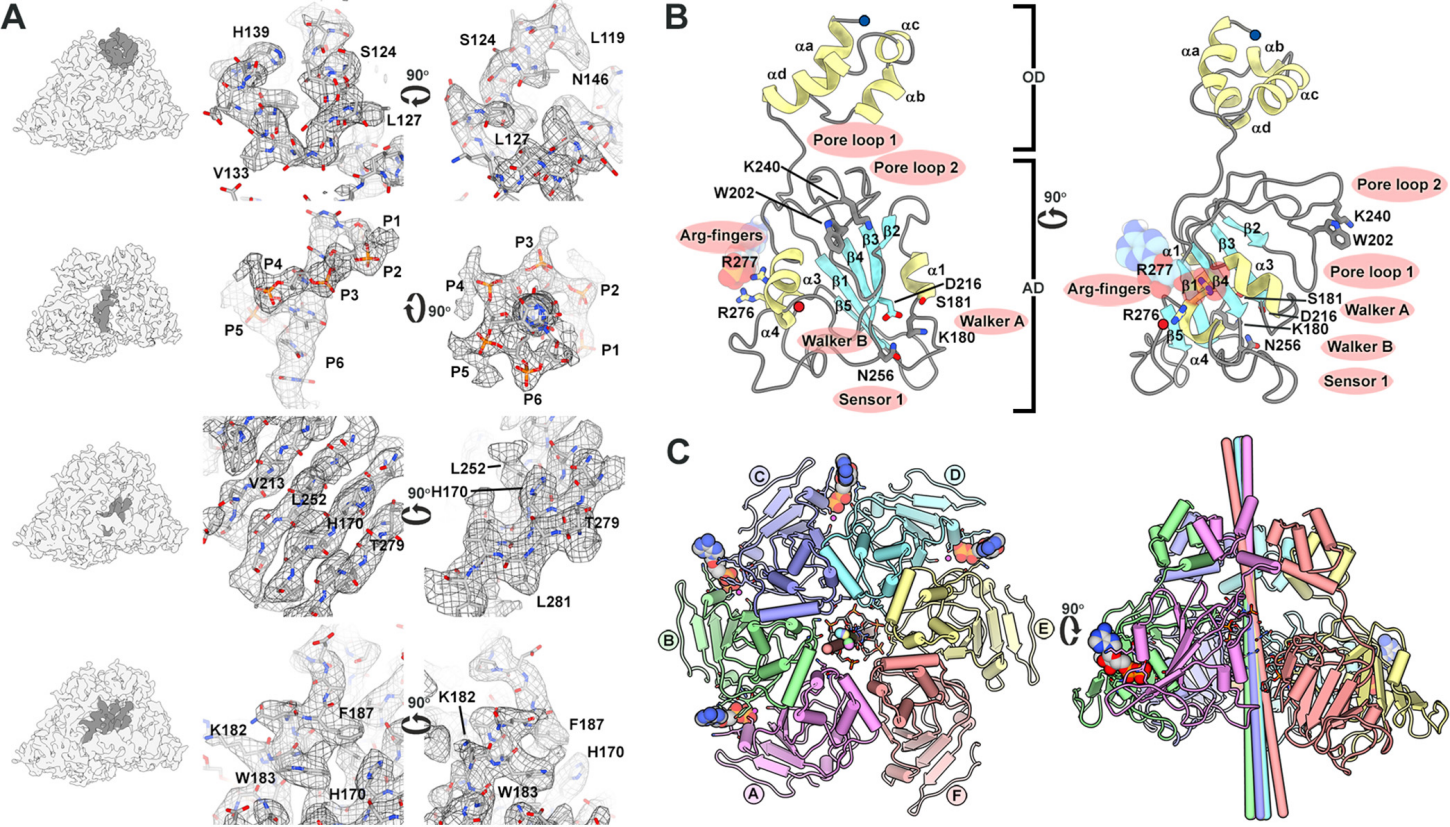
Structure of PCV2 Rep-ssDNA. (A) Modeling of coordinates into the Rep-ssDNA cryo-EM map. (Left) Cryo-EM map with modeled regions colored in dark gray. (Center) Map shown as a chicken wire mesh with model as stick representation. (Right) Ninety-degree rotation of center orientation; note the arrow for direction of rotation. (B) Ribbon-stick-CPK representation of subunit B with helices in yellow, strands in cyan, and loops in gray. The *OD* and *AD* have been labeled. Functionally important residues are identified as thick sticks. ADP is shown as CPK. The N and C termini are indicated by blue and red circles. Image on right is 90° rotation; note the arrow for direction of rotation. Image generated using UCSF Chimera X. (C) Pipe-and-plank cartoon of hexamer with subunits labeled. Subunits colored as in Fig. 1. The cylinders in the center identify the axis of rotation for overlaying the *AD* of one subunit onto the subsequent subunit. The color of the cylinder identifies the subunit that is to be rotated. The stick model ssDNA can be seen in the center of the hexamer. Figure generated using UCSF Chimera.

The cryo-EM data produce a number of distinct maps that visualize important properties of Rep (Fig. 1C and D; Tables 1 and 2). Close inspection of the two maps identifies potential boundaries for the subunits and domains. Moreover, the maps suggest that the *AD* may adopt distinct positions with respect to the *OD* (Fig. 1E). We begin by interpreting the highest-resolution map to generate an atomic model of Rep (see Fig. S1 in the supplemental material). The 3.8-Å map was manually interpreted using Coot (Fig. 2) (29). We took advantage of the high frequency (17%) of large amino acids (Arg, Phe, Trp, and Tyr) to eliminate amino acid out-of-register errors (Fig. S2) (30). The first 119 amino acids could not be modeled due to uninterpretable density. This sequence defines the *ED*, for which both NMR and X-ray crystal structures have been reported (18, 19). 3D variability analysis with cryoSPARC demonstrates this region to be mobile, adopting distinct positions in space (Fig. S3). The amino acids modeled into the cryo-EM map include the contiguous region of Leu119 to Leu301 (Fig. 2A to C). Thirteen amino acids at the C terminus of Rep could not be modeled due to poorly resolved and uninterpretable density. Water molecules were not modeled due to the modest resolution of the map.

**TABLE 1:** Cryo-EM data collection statistics^*a*^

Parameter	Replicase (ss/dsDNA)
High tension (kV)	300
Cs (mm)	2.7
Spot size	7
Detector	Gatan K2
Mode	Counting
Energy filter	No
Magnification	29,000
Pixel size	0.832 Å
No. of frames	40
Defocus range (μm)	0.3–2.4
Total dose (e^−^ Å^−2^)	71.8 e^−^ Å^2^
Dose rate (e^−^ Å^−2^ s^−1^)	7.0 e^−^ Å^2^ s^−1^
Exposure time (s)	6.0 s
Frame rate (ms)	150
No. of micrographs	5,039
Particles extracted	1,150,639
Tilts	0º
Dimension	3,710 × 3,838

a Data were collected in two sessions using the same microscope with identical parameters.

**TABLE 2:** Model refinement and validation statistics^*a*^

	Rep (ssDNA)	Rep (dsDNA)
Particles used for reconstruction	169,830	43,282
Box size	256 × 256	256 × 256
Pixel size	0.832	0.832
FSC resolution (Å)^*b*^	3.7	4.0
Global resolution (Å)^*c*^	3.8	4.4
B-factor sharpening (Å^2^)^*d*^	−207.1	−250.0
CC (model to map fit)	80.8	75.2

Model composition		
Nonhydrogen atoms	17,553	18,067
Residues		
DNA	6	24
Protein	1,092	1,092
B-factor avg (nucleotide)	58.53	157.81
B-factor avg (protein)	65.21	81.50

Root mean square deviations		
Bond lengths (Å)	0.010	0.009
Bond angles (°)	1.51	1.38
Chirality (°)	0.06	0.06
Planarity (°)	0.008	0.008

Validation		
Clashscore	4.58	4.65
* Protein validation*		
MolProbity score	1.89	1.91
EMringer score	2.85	2.29
Ramachandran plot (%)		
Favored	98.53	98.53
Allowed	1.47	1.47
Outliers	0	0
Rotamer outliers (%)	7.44	7.55
PDB entry	7LAR	7LAS
EMDB	23249	23250

a The difference in FSC and global resolution (as determined by 3DFSC) indicates minimal anisotropy. The refinement statistics are indicative of a structure with appropriate geometry.

b Fourier shell correlation reported by cryoSPARC V2 using the gold standard method at a correlation coefficient of 0.143.

c Determined by 3DFSC.

d The B-factor sharpening applied by cryoSPARC V2 during the postrefinement process.

10.1128/mBio.00763-21.1 FIG S1 Cryo-EM studies of PCV2 Rep. (A) Representative micrograph of collected data identifying different orientations of Rep. The image has been low pass filtered to 15-Å resolution to enhance the contrast. Scale bar at bottom left is 20 nm. (B) Fourier shell correlation curves reported by cryoSPARC. (C) Resolution heat maps of PCV2 Rep bound to ssDNA (top) and dsDNA (bottom). Multiple slices of the side view demonstrate the local resolution of the maps. Note that both the ssDNA and dsDNA have lower resolution than the protein. Scale bar on left identifies the resolution corresponding to the heat map. Download FIG S1, TIF file, 2.7 MB. Copyright © 2021 Tarasova et al. 2021 Tarasova et al. https://creativecommons.org/licenses/by/4.0/ This content is distributed under the terms of the Creative Commons Attribution 4.0 International license.

10.1128/mBio.00763-21.2 FIG S2 Sequence alignment of circovirus Reps. Sequence alignment generated by Clustal Omega. Image generated using ESPript 3.0 with the % equivalent and normal coloring scheme. Boxes at the top identify *ED* (pastel yellow), *OD* (pastel red), and *AD* (pastel cyan). The secondary structures of the *ED* crystal structure (PDB entry 5XOR), *OD*, and *AD* (this report) are shown inside the domain boxes. Absolutely conserved amino acids are white font in a red box with blue outline. Highly conserved amino acids are red font in a white box with blue outline. Amino acids absent in a sequence are indicated by periods. The black circles identify amino acids with large side chains (F, R, W, Y) that were used for eliminating possible register errors due to the moderate resolution of the map. Download FIG S2, TIF file, 1.4 MB. Copyright © 2021 Tarasova et al. 2021 Tarasova et al. https://creativecommons.org/licenses/by/4.0/ This content is distributed under the terms of the Creative Commons Attribution 4.0 International license.

10.1128/mBio.00763-21.3 FIG S3 3D variability analysis of the *ED*. The *ED*, *OD*, and *AD* are colored as according to Fig. 1 and Fig. S1. 3D variability manages to identify low-resolution maps of some *ED* in the data. The low number of particles in each class did not allow for a high-resolution map to be determined. Download FIG S3, TIF file, 0.8 MB. Copyright © 2021 Tarasova et al. 2021 Tarasova et al. https://creativecommons.org/licenses/by/4.0/ This content is distributed under the terms of the Creative Commons Attribution 4.0 International license.

The modeled coordinates define two domains: an *OD* (amino acids 119 to 157) and an *AD* (amino acids 158 to 301). The *OD* is a four-helix bundle with three of the helices nearly parallel to one another and the fourth perpendicular to them. Six *OD* oligomerize to generate a torus with a pore diameter of 12 Å. The pore is defined by the side chains of Arg145, Asn146, Tyr147, and Arg148. Segmentation of density pertaining to the *OD* hexamer followed by 60, 120, 180, 240, and 300° rotations through the center of the pore overlays onto the original density with correlation coefficient values between 0.95 and 1.0, suggesting that the *OD* hexamer follows C6 symmetry. Sequence comparison suggests that all circovirus Reps share a minimum of 30% sequence identity in this region; thus, it is likely that all circovirus Reps utilize a comparable *OD* for forming hexamers (Fig. S2).

The *OD* is connected to the *AD* via a seven-amino acid loop that adopts a distinct conformation for each subunit. The *AD*s are arranged like a spiral staircase. We will refer to the six subunits as A through F, with the *AD* of subunits A and F located at the top and bottom of the staircase, respectively. The *AD* adopts an αβα-fold, where five-parallel β-strands define a β-sheet with two α-helices on one face and one α-helix on the opposite face (Fig. 2B and C). A Dali search with the *AD* identifies the ATPase domain of the enterovirus 71 2C helicase (EV71, PDB entry 5GRB), the simian virus 40 (SV40) large T antigen (LTag, PDB entry 1N25), adeno-associated virus 2 (AAV2) Rep40 (PDB entry 1S9H), and bovine papillomavirus (BPV) E1 (PDB entry 2GXA), all members of SF3 from the AAA+ superfamily (31). Hallmarks of SF3 ATPases include the Walker A (WA, Lys180 located between β1 and α1) and Walker B (WB, Asp216 located between β3 and α3) motifs, motif B (mB, Lys240 and Gly241 located between α3 and β4), motif C or sensor 1 (mC, Asn256 following β4), sensor 2 (Arg276), and sensor 3 (Arg228) (Fig. 2B) (32, 33).

The six *AD* overlay with root mean square deviation (RMSD) values in the range of 0.7 to 1.1 Å. The arrangement of *AD* can be described by six rotation axes about which a rotation followed by a translation, parallel to the axis, will overlay two neighboring *AD*s (Fig. 2C; Table 3). Comparison of these values demonstrates that the hexamer is asymmetric and draws attention to the distinct nature of the *AD*-*AD* interfaces. Between each of five neighboring *AD* (subunits A to E), there is 760 Å^2^ of buried surface area (BSA). An extensive seam between the subunits F and A diminishes their *AD* interaction to 80 Å^2^ of BSA (Fig. 2C). The BSA, number of hydrogen bonds (H-bonds), and number of salt bridges increase as one steps away from the seam to reach a maximum at the interface distal to the seam (subunits CD). For subunit A, where *AD* is at the apex of the staircase, amino acids 129 to 133 of *OD* interact with amino acids 206 to 210 of *AD*.

**TABLE 3:** Comparison of PCV2 Rep and BPV E1 architecture^*a*^

Overlay^*b*^	Rotation^*c*^ (º)		Translation^*d*^ (Å)		Offset^*e*^ (º)	
	PCV2	BPV	PCV2	BVP	PCV2	BPV
A → B	58.5	53.1	2.5	2.2	7.7	7.5
B → C	59.2	57.9	2.7	−1.5	8.9	5.8
C → D	58.6	57.0	3.1	−1.8	11.1	7.1
D → E	59.7	63.4	3.1	−3.3	11.9	8.6
E → F	60.0	63.7	3.2	−2.6	10.5	5.5
F → A	64.1	65.6	−14.7	7.5	16.2	11.3

a Parameters for overlaying an *AD* subunit onto the *AD* of a neighboring subunit. Values are shown for the PCV2 Rep and BPV E1 (PDB entry 2GXA). The large difference in the direction of translation between Rep and E1 is indicative of a potential difference in DNA translocation mechanism. Note that the Rep *AD* continues to move away from the *OD*, whereas the E1 *AD* oscillates from the *OD*.

b Alignment of AD from one subunit to the neighboring subunit.

c Relative rotation needed to align the *AD*.

d Relative translation (shift) along the axis shown in Fig. 2. Positive values indicate movement away from the *OD*.

e The offset degree from the *OD* C6 axis of rotation. The direction of offset is shown in Fig. 2.

### Nucleotide binding states of Rep suggest a sequential mode of ATP hydrolysis.

**FIG 3:**
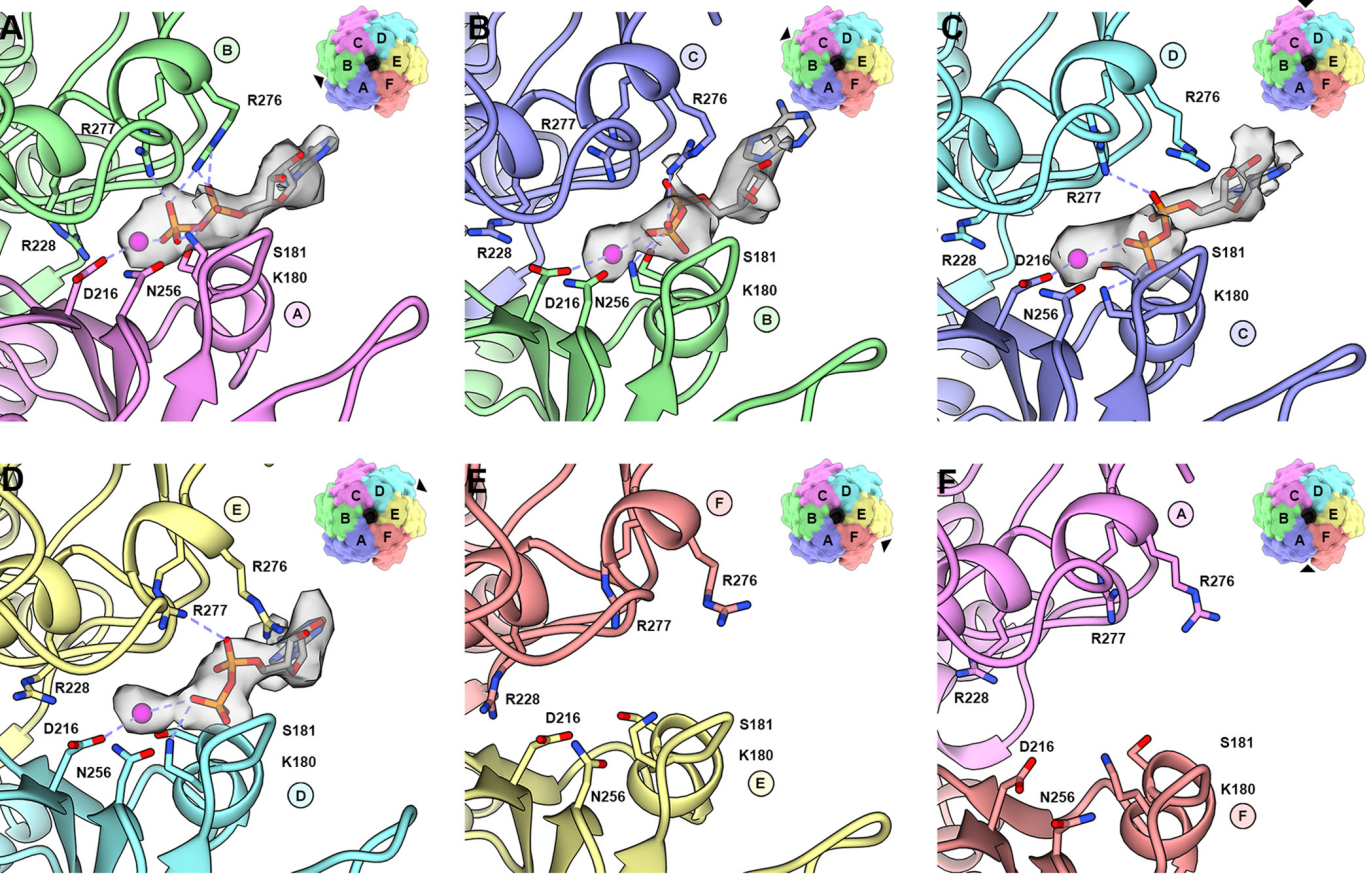
Nucleotide binding pockets of Rep-ssDNA structure. Ribbon-and-stick cartoon showing the bound ADP to interfaces AB, BC, CD, and DE. Hydrogen bonds and electrostatic interactions are shown as dashed lines. The ADP is modeled as a gray stick model, its corresponding density as a transparent surface mesh, and the Mg^2+^ as a purple sphere. The surface representation on the top right of each panel highlights the nucleotide binding site (arrowhead) with respect to the hexamer. (A) AB subunit interface (ATP-like); (B) BC subunit interface (ATP-like); (C) CD interface (ADP); (D) DE interface (ADP); (E) EF interface (empty); (F) FA interface (empty). Images generated using UCSF Chimera and UCSF Chimera X.

The ATP binding sites for members of SF3 ATPases are defined by two neighboring subunits. One subunit provides the *cis*-components WA, WB, mB, and mC while the neighboring subunit provides the *trans*-components sensor 2 (Arg-finger), and sensor 3. Density not described by the Rep coordinates can be observed within four of the putative ATP binding sites (Fig. 3A to D). The densities are situated at the interface of subunits AB, BC, CD, and DE. We interpret these densities to represent ADP and Mg^2+^ for two reasons: (i) ATP-Mg^2+^ was added during purification, and (ii) Rep is an ATPase (see below) and likely to have hydrolyzed the ATP present in the buffer. Into each of the four densities could be modeled a Mg^2+^, two phosphates, and the ribose sugar of ADP (Fig. 3A to D). Protein-ADP-Mg^2+^ interactions common to all binding sites include H-bonds between main chain amides of Gly179, Lys180, and Ser181 (WA) and the ADP β-phosphate, an H-bond between the Lys180 (WA) amine and ADP β-phosphate, electrostatic interaction between the Asp216 carboxylate (WB) and Mg^2+^, and electrostatic interaction between Mg^2+^ and the ADP phosphates. Also, the Asn256 (mC) amide is in proximity to position a water for nucleophilic attack on the ATP γ-phosphate (Fig. 3). Interactions unique to each interface may correspond to the nucleotide binding state; these include (i) H-bonds between Arg276 and Arg277 guanidinium and the ADP β-phosphate in the AB interface (Fig. 3A and Fig. S4A), (ii) H-bonds between Arg276 guanidinium and the ADP β-phosphate in the BC interface (Fig. 3B and Fig. S4B), and (iii) H-bonds between Arg277 guanidinium and the ADP α-phosphate in the CD and DE interfaces (Fig. 3C and D and Fig. S4C and D). In total, there are seven direct interactions between Rep and ADP in the AB interface, three in the BC interface, two in the CD and DE interface, and no nucleotide present in the EF or FA interfaces. Each ADP contributes 200 Å^2^ of BSA to the protein-nucleotide interaction. The decrease in interaction between Rep-ADP, as one steps through the interfaces descending down the staircase, suggests that the latter interfaces may describe later stages of ATP hydrolysis, a model consistent with sequential ATP hydrolysis.

10.1128/mBio.00763-21.4 FIG S4 Nucleotide binding states of Rep. (A to D) (Top) Licorice models with the *cis*-subunit in yellow and *trans-*subunit in red. (Bottom) LigPlot+ images of Rep-ADP interaction. Residues involve in hydrophobic contacts are depicted as arcs with emanating lines, and hydrogen bonds are depicted as dashed lines. (A) Subunit interface AB; (B) subunit interface BC; (C) subunit interface CD; (D) subunit interface DE; (E) ATP state of SV40 LTag; (F) ADP state of SV40 LTag; (G) ATP-like state of BPV E1; (H) ADP state of BPV E1. The Mg^2+^ are shown as magenta spheres, and the Cl^−1^ of BPV E1 is shown as a larger pink sphere. Download FIG S4, TIF file, 1.2 MB. Copyright © 2021 Tarasova et al. 2021 Tarasova et al. https://creativecommons.org/licenses/by/4.0/ This content is distributed under the terms of the Creative Commons Attribution 4.0 International license.

We compared the nucleotide binding mode of Rep to those of SV40 large T antigen (LTag) and BPV E1 by overlaying subunits providing the *cis*-components (Fig. S4). LTag and E1 possess two Arg amino acids and one Lys amino acid that interact with the phosphates of nucleotides (33, 34). These amino acids overlay in the ATP-bound LTag (PDB entry 1SVM) and ADP-bound E1 (PDB entry 2GXA) structures. The Arg498 of LTag coordinates a water molecule to the ATP γ-phosphate and is proposed to act as a switch in sensing ATP binding and coordinating its hydrolysis to the neighboring subunit (34). Arg498 also interacts with Asp474 (WB) and Asn529 (sensor 1). Equivalent amino acids in E1 include Arg493, Asp479, and Asn523. The Arg-finger of LTag (Arg540) directly stabilizes the ATP γ-phosphate, whereas the Arg-finger of E1 (Arg538) interacts with a chloride ion that is positioned where the ATP γ-phosphate is anticipated to be. There is no ATP-bound structure of E1. The third member of this triad, Lys418 of LTag and Lys425 of E1, interacts with the β- and α-phosphates of the nucleotides. Amino acids from Rep that overlay with the Arg-Arg-Lys triad include Arg228 (LTag/E1: Arg498/Arg493), Arg277 (Arg540/Arg538), and Arg276 (Lys418/Lys425). However, the Rep triad exhibits slightly different interactions with the bound nucleotide that include the following: (i) Arg228 is dissociated from the ADP nucleotide, but is positioned properly to interact with the γ-phosphate of an ATP, and (ii) one or both of Arg276 and Arg277 are engaged with the ADP β- and α-phosphate. The most significant differences between the nucleotide binding pockets of LTag, E1, and Rep include the greater separation between the A and F subunits of Rep and the extent to which the nucleotides of Rep are exposed to solvent. The C termini of LTag and E1 further interact with the adenosine base of the nucleotide, forming at least one H-bond and burying 100 Å^2^ of surface area. Similar extensions are also present in the structures of AAV2 Rep40 and EV71 2C (Fig. S5).

10.1128/mBio.00763-21.5 FIG S5 The lid-like domains of SF3 helicases. Cartoon ribbon model of the ATPase domains in red, C-terminal extension (lid-like domain) in yellow, and nucleotide in CPK. (A) SV40 LTag bound to ADP (1SVM); (B) EV71 2C bound to ATP-γ-S (5GRB); (C) AAV2 Rep40 bound to ADP (1U0J); (D) BPV E1 bound to ADP (2GXA); (E) PCV2 Rep bound to ADP (this report). The N and C termini are indicated by blue and red circles. Download FIG S5, TIF file, 1.1 MB. Copyright © 2021 Tarasova et al. 2021 Tarasova et al. https://creativecommons.org/licenses/by/4.0/ This content is distributed under the terms of the Creative Commons Attribution 4.0 International license.

### Rep is an ATPase with basal NTPase activity.

**FIG 4:**
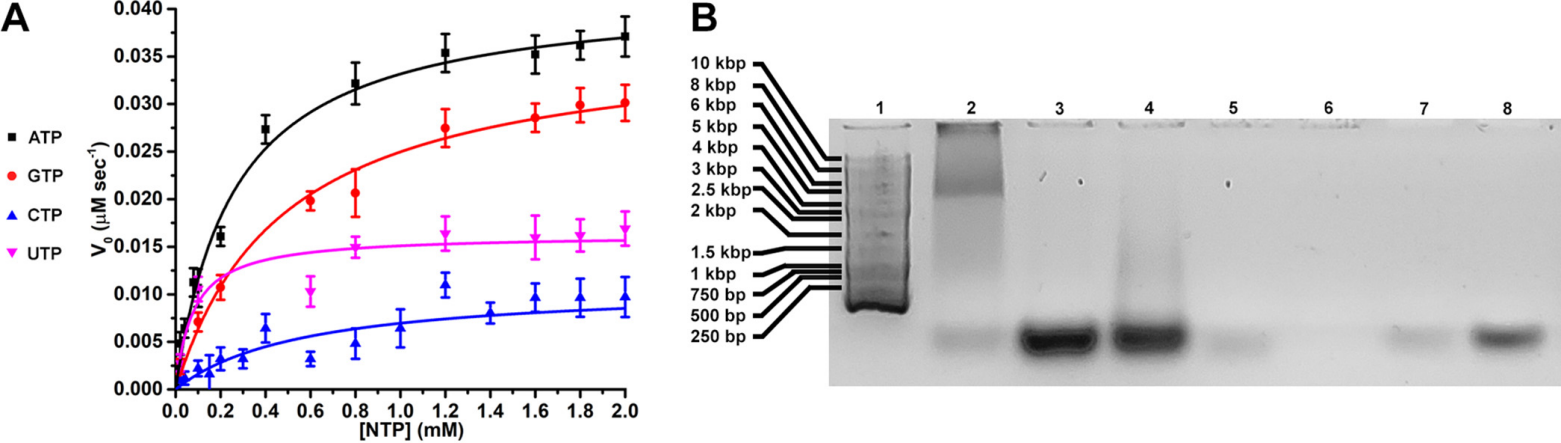
Functional studies of PCV2 Rep. (A) The basal NTPase activity of Rep was measured using the ATP/NADH-coupled spectrophotometric assay under Michalis-Menten conditions. Rep can hydrolyze ATP, GTP, CTP, and UTP, with a preference for ATP. (B) Agarose gel of Rep demonstrating that it binds exclusively to DNA. Lane 1, 1-kbp ladder (Gold Biotechnology); lane 2, Rep at room temperature; lane 3, denatured Rep (dRep); lane 4, dRep treated with RNase I (2 h); lane 5, dRep treated with DNase I (1 h); lane 6, dRep treated with DNase I (2 h); lane 7, 12-nt ssDNA; lane 8, 44-nt ssDNA.

The cryo-EM map of Rep visualizes density for the Mg^2+^, the ADP pyrophosphate, and ribose; however, the density for the adenosine base is limited and weak (Fig. 3). The lack of interaction between the modeled adenosine and Rep suggests that Rep may not be selective toward the base of nucleotides. To address this possibility, we measured the basal NTPase activity of Rep using an NTP/NADH-coupled spectrophotometric assay under Michaelis-Menten conditions (35). Titration of ATP onto 66 nM Rep demonstrated that Michaelis-Menten kinetics is observed (Fig. 4A). To ensure that Rep is indeed an ATPase, we tested the ATPase activity of Lys180Ala (WA), Asp216Ala (WB), and Asn256Ala (mC) variants using the same assay. Indeed, the assays demonstrate that these substitutions diminish ATP hydrolysis to undetectable levels, values comparable to equivalent substitutions in other ATPases (Table 4) (36). This demonstrates that Rep is indeed an ATPase. We then titrated increasing concentrations of GTP, CTP, and UTP onto 66 nM Rep to see if Rep demonstrates NTPase activity. Indeed, increasing concentrations of NTP resulted in increasing rates of NADH oxidation to NAD^+^ (Fig. 4A).

**TABLE 4:** Enzymatic activity of wild type and variants of Rep^*a*^

Nucleotide	*k*_cat_ (s^−1^)	*S*_0.5_ (μM)
ATP	0.63	261.1
GTP	0.56	577.2
CTP	0.16	284.4
UTP	0.24	78.5
Mutant	*k*_cat_ (s^−1^)	*S*_0.5_ (μM)
Lys180Ala	ND	ND
Asp216Ala	ND	ND
Asn256Ala	ND	ND

a Assays performed under Michaelis-Menten conditions using the ATP/NADH-coupled assay. Total Rep concentration used per reaction is 66 nM. We use the term *S*_0.5_ to denote that ATP hydrolysis by Rep may not necessarily exhibit Michaelis-Menten kinetics. This needs further attention in future studies. The activities of WA, WB, and motif C variants were also measured. ND, activity not detected.

### Rep uses two loops to recognize the phosphates of single- and double-stranded DNA.

Density can be seen in the center of the Rep hexamer spiraling away from the *OD* (Fig. 2A). Biochemical data have shown that Rep can unwind dsDNA in a 3′-to-5′ direction (19). We hypothesized that the material bound to Rep could be nucleic acid. To test for the presence of nucleic acid, we processed 0.4 nmol of Rep using 1% agarose and stained with SYBR-Gold (Thermo Fisher) (Fig. 4B). Indeed, fluorescence of SYBR-Gold suggests that nucleic acid material may be present in the sample, an agreement with the 260/280-nm ratio. We then heat denatured Rep so that it releases the bound material and digested the material with either RNase I or DNase I (Thermo Fisher). The results demonstrate that heat denaturation results in increased fluorescence by SYBR-Gold, and treatment with DNase I, but not RNase I, results in abolished fluorescence (Fig. 4B), suggesting that DNA is present in the Rep sample. To determine if DNA can be modeled into the cryo-EM map, we generated a poly(dT) B-DNA using the x3DNA server (37) and manually docked it into the mentioned density using UCSF Chimera (38). The docking reveled that the density could accommodate only ssDNA. To determine which orientation of ssDNA can bind to Rep, we divided the dsDNA into two ssDNAs and manually docked each strand (orientation) into the density as a rigid body. We then trimmed each ssDNA to six bases and refined the coordinates using the ProSMART Generate All-Molecule Self Restraints 4.3 tool of Coot and the real space refinement tool of Phenix (39, 40). The 3′-to-5′ orientation refined to a cross correlation (CC) of 0.64 with a Clashscore of 0.0, and the 5′-to-3′ orientation refined to a CC of 0.5 with a Clashscore of 4.4. The better quality of fit for the 3′-to-5′ orientation independently agrees with the biochemical data demonstrating Rep to exhibit 3′-to-5′ helicase activity (19). The model suggests that Rep translocates along ssDNA with its *ED* leading the charge. While the density describing the sugar-phosphate backbone of the ssDNA is strong and convincing, the density describing the bases of the nucleic acid is smeared, likely due to conformational and sequence heterogeneity within the data set, and therefore, the modeled poly(dT) is a generalized interpretation of the multiple ssDNA sequences bound to Rep.

**FIG 5:**
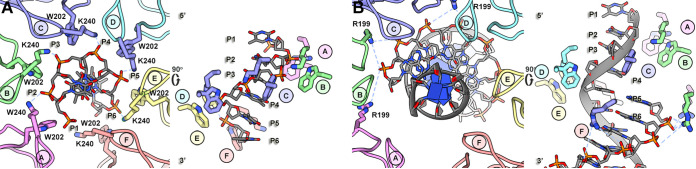
Rep DNA interaction. (A) (Left) Ribbon-stick cartoon of Rep in complex with ssDNA. The six subunits of Rep are colored and labeled according to Fig. 1. Amino acids Trp202 (pore loop 1) and Lys240 (pore loop 2) are shown as sticks. The interactions between Lys240 and ssDNA are shown as dashed lines. (Right) Stick cartoon of ssDNA and Rep rotated by 90°. The 5′-PO_4_ and 3′-OH of ssDNA have been labeled, and the spiral staircase arrangement of Trp202 and Lys240 is demonstrated. (B) (Left) Ribbon-stick cartoon of Rep interacting with dsDNA. The interactions described for Rep-ssDNA persist. Image focuses on the interaction between Rep and the second strand of DNA—identical orientation as shown in panel A. (Right) Ninety-degree rotation of model on left. The side chains for Trp202 and Lys240 are shown. The interactions between Arg199 and the second strand of DNA are shown as dashed blue lines.

The backbone phosphates from four consecutive nucleotides interact with the six subunits of Rep (1 nt subunit^−1^). Density for the ssDNA extends toward the center of the *OD* ring and terminates near the guanidium group of Arg148, suggesting that ssDNA is translocated through the pore. Rigid body fitting of a purine nucleotide into the pore suggests that its translocation requires either the dehydration of the nucleotide, rotation of the base, or expansion of the pore. The phosphates from ssDNA interact with amino acids located in two loops: pore loop 1 located between β2 and α2 (Trp202) and pore loop 2 (also known as the “presensor 1 β-hairpin”) located between α3 and β4 (Lys240 and Gly241) (Fig. 2B and Fig. 5A). The ssDNA phosphate (P1) at the top of the staircase makes no interaction with Rep. The subsequent phosphate (P2) forms an H-bond to the Lys240 amine of subunit A. Subunit B also donates an H-bond (amide of Gly241) to P2. Sandwiched between P2 and P3 is the indole of Trp202 from subunit B. The amine of Lys240 from subunit B forms an H-bond to P3. This set of interactions is continued until subunit F, where no interaction between the subunit and the ssDNA is observed.

Heterogeneous classification identifies dsDNA bound to the center of Rep (Fig. S1C). Using the x3DNA web server, we generated A-DNA, B-DNA, C-DNA, and RNA models of 36 nucleotides (per strand) and then computationally docked each into the segmented map of the nucleic acid using MOLREP, Phenix, and UCSF Chimera (Fig. S6) (37–39, 41). The fits of the B- and C-DNA into the segmented map are comparable and substantially better than A-DNA or ssRNA (Table 5). Given the comparable fits of B- and C-DNA and the fact that B-DNA is anticipated to be the more biologically relevant form, we modeled B-DNA with a 5′ hexanucleotide overhang extending into the *OD* pore. The 5′ overhang agrees with the 3′-to-5′ directionality of Rep. In addition to the described interactions between Rep and the ssDNA, the guanidinium groups of Arg199 (pore loop 1) from subunits A to C are less than 7.5 Å from the first four nucleotide phosphates of the second strand of nucleic acid (Fig. 5B). This distance is close enough for electrostatic interaction (42). Arg199 is highly conserved among *Circoviridae* (Fig. S2).

**TABLE 5:** Fitting of dsDNA into cryo-EM map of Rep^*a*^

DNA type	CC_mask	CC_volume	CC_peaks	CC_box
A-DNA	0.35	0.29	−0.12	0.64
B-DNA	0.66	0.62	0.23	0.81
C-DNA	0.65	0.60	0.23	0.80
RNA	0.21	0.12	−0.24	0.57

a Cross correlation (CC) values of models objectively fitted into the cryo-EM map using the real space fitting and refinement of Molrep. CC values were calculated using phenix.validation_cryoem.

10.1128/mBio.00763-21.6 FIG S6 Structure of PCV2-dsDNA. Modeling of coordinates into the Rep-dsDNA cryo-EM map. (Left) Cryo-EM map with modeled regions colored in dark gray. (Center) Map shown as a chicken wire mesh with model as stick representation. (Right) Ninety-degree rotation of center orientation; arrow identifies direction of rotation. The dsDNA map (second from top) is low pass filtered to demonstrate the fit of the double helix. This region has a much lower resolution than the protein; see Fig. S1. Download FIG S6, TIF file, 2 MB. Copyright © 2021 Tarasova et al. 2021 Tarasova et al. https://creativecommons.org/licenses/by/4.0/ This content is distributed under the terms of the Creative Commons Attribution 4.0 International license.

## DISCUSSION

CRESS-DNA viruses are widely distributed in nature and utilize RCR for genome replication (3, 6). While studies have provided insight into the general mechanism of RCR, little information is available on the structural mechanism of RCR (4). A recent crystal structure of the PCV2 *ED* in complex with a 10-mer ssDNA derived from the viral *ori* provides the first image of how *ED* binds to the loop of the predicted *ori* stem-loop hairpin for the nicking activity described above (43). To provide structural insight into the lacking information, we determined the first structure of a CRESS-DNA Rep (Fig. 1). The structure identifies a hexameric molecule composed of three domains: (i) the known *ED* at the N terminus, (ii) an *OD* in the middle of the sequence, and (iii) an SF3 *AD* at the C terminus (Fig. 1). The density associated with the *ED* is indistinguishable in the 3.8-Å cryo-EM map, and 3D variability analysis with cryoSPARC reveals the *ED* to adopt multiple positions in space (see Fig. S3 in the supplemental material). It remains to be determined if the interaction between *ED* and *ori*, *AD* and nucleotide (ATP/ADP/empty), and Rep with no DNA affects this property. Six ODs assemble to form a torus with a pore defined by a 12-Å diameter. The *AD* structure is homologous to the ATPase domains of SV40 LTag, the BPV E1, the AAV2 Rep40, and the EV71 2C helicase proteins.

The six ADs arrange into a staircase that spirals away from the OD, with subunits A and F at the top and bottom of the staircase, respectively (Fig. 2C). Four ADP molecules are bound to Rep, between subunits AB, BC, CD, and DE (Fig. 2C and Fig. 3). The nucleotide binding sites are defined by WA (Lys180), WB (Asp216), mB (Lys240 and Gly241), mC or sensor 1 (Asn256), sensor 2 (Arg276), and sensor 3 (Arg228). The extent of interaction (BSA, H-bonds, and salt bridges) between Rep and ADP diminishes as one descends the staircase, suggesting that the interactions near the top of the staircase may be representative of Rep-ATP and interactions near the bottom of the staircase may be representative of Rep-ADP. We propose that interfaces AB and BC bind ATP, interfaces CD and DE bind ADP, and interfaces EF and FA are empty. Substitution of the WA, WB, and sensor 1 amino acids abrogates Rep’s basal ATPase activity (Table 4). It would be interesting to see if such substitutions affect the viability of PCV2 under cellular and *in vivo* conditions. Rep binds ssDNA in a 3′-to-5′ direction, such that the Rep N terminus is positioned near the dsDNA fork and ssDNA is pulled through the *OD* pore (Fig. 5). Rep subunits bind to ssDNA using three amino acids: the indole of Trp202 (sandwiched between two ssDNA phosphates and juxtaposed to the ribose), the amine of Lys240 (H-bonding to the ssDNA phosphates), and the amide of Gly241 (H-bonding to the ssDNA phosphates) (Fig. 5).

**FIG 6:**
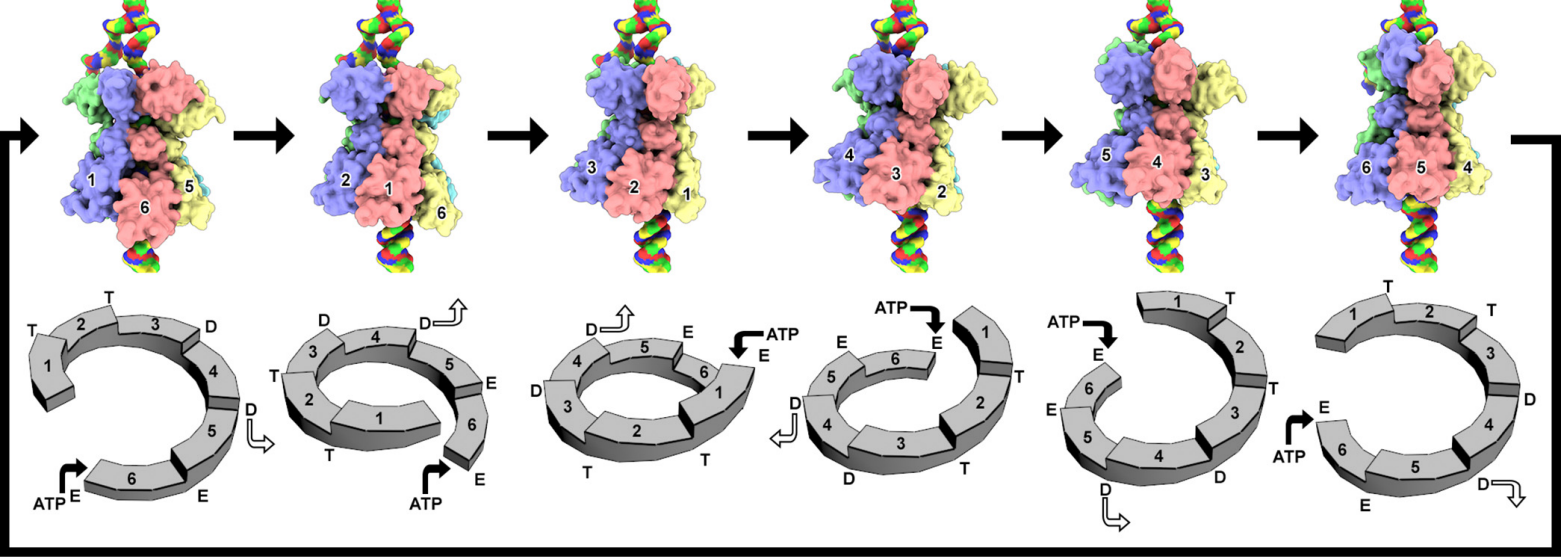
Translocation of ssDNA by Rep. The six images on top demonstrate the translocation of each Rep *AD*. The six images on the bottom identify the location of the *AD* within the staircase arrangement. Rep subunits move from one position to the next to translocate ssDNA (6 → 1, 1 → 2, 2 → 3, 3 → 4, 4 → 5, and 5 → 6). Subunits A to F are color coded according to Fig. 1. The dsDNA fork is shown at the top, with the displaced ssDNA moving to the left. dsDNA is shown at the bottom of Rep, where its 3′-OH serves as the DNA elongation site during replication. Attention is given to the subunits A (pastel magenta), F (pastel red), and E (pastel yellow). The Arabic numbers identify the position of each subunit on the staircase. Binding of ATP to the nucleotide binding site of the subunit at position 6 (bottom of staircase) promotes its translocation to position 1 (top of the staircase). Consequently, each subunit moves to the next position in the staircase. Binding of ATP to the 6-1 interface and release of ADP from the 4-5 interface are shown at the bottom using filled and unfilled arrows. Each step translocates Rep by ∼3.5 Å toward the dsDNA fork.

Two models proposed for the translocation of biological macromolecules through the central channel of ring-shaped oligomeric ATPases are the sequential and concerted models. The sequential model was first proposed for the catabolism/anabolism of ATP by the F1-ATPase heterohexamer (32, 44). The F1-ATPase has three ATP binding sites. One site is occupied by ATP, the other is occupied by ADP + P_i_, and the third is empty. The F1-ATPase catabolizes ATP as each site cycles through the three nucleotide states (e.g., ATP → ADP + P_i_ → empty → ATP). ATP anabolism is the reverse of this process. The sequential model was adopted to describe the translocation of nucleic acid by the homohexameric T7 gp4 and Rho (45, 46). For these hexamers, two sequential nucleotide binding sites are occupied by ATP, two sequential sites are occupied by ADP, and two sequential sites are empty (47, 48). Structural studies of the BPV E1 homohexamer in complex with ssDNA demonstrated the *AD*s to adopt a staircase arrangement around the nucleic acid and identified ADP bound to the four *AD*s at the top of the staircase (33). Enemark and Joshua-Tor (33) proposed that sequential binding and hydrolysis of ATP resulted in rigid body movements of E1 *AD* that are responsible for translocating the ssDNA through the central channel. Shortly thereafter, a similar mechanism was proposed for the translocation of RNA through the central channel of Rho (49). Consistent with the sequential model are structural studies from several hexameric AAA+ proteins that are responsible for eukaryotic protein quality (50). The theme emerging from these studies is as follows: (i) the *AD*s adopt a staircase arrangement around their biopolymer substrate; (ii) the nucleotide states for the six *AD*s from the top to bottom of the staircase are ATP, ATP, ATP/ADP, ATP/ADP, ADP/empty, empty; (iii) only the central four *AD*s engage the substrate; (iv) the *AD* at the bottom of the staircase translocates to the top of the staircase upon binding ATP; (v) hydrolysis of ATP for the *AD* closest to the bottom of the staircase drives the remaining *AD*s to translocate toward the bottom of the staircase and pull the substrate; and (vi) ADP is released from the *AD* that is nearest to the bottom of the staircase. Consequently, each ATP binding and hydrolysis translocate the substrate one unit through the central channel of the translocase (Fig. 6). The concerted model was first proposed by Gai et al. (34) as the mechanism of ssDNA translocation by the SV40 LTag helicase. This mechanism was deduced from crystal structures of LTag homohexamers bound to six ATP or six ADP or nucleotide free. The structures demonstrated conformational differences between LTag in the different nucleotide states, yet all adopted a planar arrangement. These observations led the authors to propose an “all-or-none” ATP binding mode followed by concerted ATP hydrolysis and ADP release for ssDNA translocation (34). The concerted model was more recently proposed for the translocation of polypeptide through the *Plasmodium* translocon of exported proteins (PTEX), a 1.6-MDa complex that transports *Plasmodium* proteins into the host erythrocytes across the Plasmodium membrane (51). Using cryo-EM, Ho et al. (51) visualized the PTEX complex bound to endogenous substrate in two states defined as engaged and resting. The *AD*s adopt a staircase arrangement around the substrate in the engaged state and organize to a planar arrangement in the resting state. The authors proposed the three *AD*s at the top of the staircase to engage and push the substrate through the central channel as the staircase collapses to the planar arrangement. These *AD*s then release the substrate and transition to the staircase arrangement to repeat the process. Consequently, PTEX uses ATP binding and hydrolysis to transition between these two states for translocating its cargo through its central channel.

The *ADs* of Rep move as rigid bodies along the ssDNA to maintain their contacts with the phosphate-sugar backbone. We anticipate Rep to translocate ssDNA through the pore defined by the six ODs. Subunits A (top of staircase, Fig. 6) and F (bottom of staircase, Fig. 6) make limited interactions with ssDNA, whereas subunits B through E more intimately engage ssDNA. We propose that in the presence of ATP, subunits A through E translocate one step toward the bottom of the staircase while subunit F translocates to the top (Fig. 6). The more intimate interaction between subunits B through E and the ssDNA suggests that it is these subunits that are responsible for pulling the ssDNA through the *OD* pore. The pulling requires hydrolysis of the ATP in the BC interface, release of ADP in the DE interface, and translocation of subunit F to the top of the staircase accompanied by binding of an ATP to the FA interface. This completes one cycle of ATP binding, hydrolysis, ADP release, and translocation of one ssDNA nucleotide to generate a structure that is comparable to the starting structure. It remains to be described what causes the *AD* of subunit F to translocate to the top of the staircase.

One important distinction between Rep and the above-mentioned SF3 helicases is a shortened Rep C terminus. The C termini of LTag, EV71 2C, Rep40, and E1 form helical structures that interact with the adenosine base of the nucleotide (Fig. S5). Indeed, biochemical studies have demonstrated that substitutions in the mentioned LTag C terminus regulate the helicases’ specificity for ATP, TTP, and UTP (52). The lack of such a domain in Rep may be responsible for the observed basal NTPase activity of Rep. It remains to be determined if Rep can melt dsDNA in the presence of NTP.

The Rep-dsDNA structure demonstrates that Rep is able to sense the second strand of dsDNA (Fig. 5 and Fig. S6). Thus, it is plausible that Rep loads onto ssDNA such that it is juxtaposed to the newly generated 3′-OH used for leading-strand synthesis. In this regard, this structure may be visualizing an earlier stage of RCR.

## MATERIALS AND METHODS

### Protein expression and purification.

The DNA sequence for PCV2 Rep (GenBank: ABM88862.1) was codon optimized by GenScript (New Jersey) and cloned into a modified pET28a vector containing a small ubiquitin modifier (SUMO) following the start codon for expression in Escherichia coli BL21(DE3) cells. Cells were grown in terrific broth in the presence of 50 μg ml^−1^ kanamycin until mid-log phase and cooled to 20°C, protein expression was induced with 200 μM isopropyl-β-d-1-thigalactopyranoside (IPTG), and protein was expressed for less than 16 h at 20°C. Cells were centrifuged at 4,000 × *g* for 30 min. Cell pellet was resuspended in 35 ml of 20 mM HEPES (pH 8.2), 450 mM sodium chloride (NaCl), 25 mM imidazole (pH 8.2), 10 mM magnesium chloride (MgCl_2_), 0.5 mM Tris(2-carboxyethyl)phosphine hydrochloride (TCEP), 100 mM phenylmethylsulfonyl fluoride (PMSF), 0.5 μl salt-activated nuclease (Millipore Sigma), 1 mM ATP (Millipore Sigma) and lysed using a sonicator. The lysate was centrifuged at 32,000 × *g* for 40 min at 4°C. The supernatant was applied to 5 ml of Ni-NTA (nitrilotriacetic acid) chromatography resin (Gold Biotechnology), washed with 5 column volumes (CV) using the same buffer, eluted, and fractionated with 5 CV of the same buffer supplemented with 600 mM NaCl and 750 mM imidazole (pH 8.2). Sodium dodecyl sulfate-polyacrylamide gel electrophoresis (SDS-PAGE, 12%) was used to visualize the protein content of the fraction. Fractions with the greatest Rep content were pooled and digested with 100 μg of Ulp1 protease for 1 h at 4°C to hydrolyze the SUMO fusion at the N terminus, diluted to 300 mM NaCl using the same buffer, and processed using a 1-ml heparin-conjugated chromatography resin (HyperD; Pall Life Sciences) connected to an Äkta Pure (GE Healthcare Life Sciences). Sample was washed with 3 CV of the same buffer and eluted using a salt gradient (0 to 2 M NaCl) in the same buffer. Fractions possessing Rep were concentrated using ultrafiltration and processed using size exclusion chromatography (Superose 6 10/300) connected to an Äkta Pure equilibrated in 20 mM HEPES (pH 8.2), 500 mM NaCl, 0.2 mM TCEP, 10 mM MgCl_2_ (GE Healthcare Life Sciences). Fractions possessing Rep were pooled, concentrated, flash frozen using N_2_(l), and stored at −80°C. Ulp1 was purified as previously described (53). Rep variants were generated using the Q5 site-directed mutagenesis kit from New England BioLabs Inc. Mutations were confirmed using the Sanger sequencing services of Genewiz (New Jersey).

### Concentration determination.

Absorption spectroscopy indicated, via a 260-nm/280-nm ratio, that despite the two steps of affinity purification under high salt concentration and size exclusion chromatography, nucleic acid copurifies with Rep; thus, protein concentration was determined using quantitative SDS-PAGE analysis. The PAGE was quantitated using the Li-Cor Odyssey blot imager equipped with Image Studio software version 5.0. The concentration of Rep (35.8 kDa) was determined by comparing its band intensity to that of carbonic anhydrase (29 kDa; Millipore Sigma) with known concentration.

### Basal NTPase assay.

The ATP/NADH-coupled spectrophotometric assay was performed using 66 nM Rep per reaction (35). Measurements were performed using a VWR UV-Vis scanning UV-1600PC spectrophotometer. Briefly, the assay was performed at room temperature in the presence of 0.42 mM phosphoenolpyruvate (Millipore Sigma), 0.15 mM NADH (Roche), 7.5 IU pyruvate kinase (Millipore Sigma), 18 IU lactate dehydrogenase (Millipore Sigma) and increasing concentrations of NTP. Absorbance at 340 nm was measured for 10 min, and the slope of the line (negative) was used to determine the absorbance unit time^−1^ (AU time^−1^) rate of NADH conversion to NAD^+^. AU was converted to concentration using a calibration curve of NADH concentration (known) versus absorbance (measured). Kinetic parameters (*k*_cat_ and *S*_0.5_) were extracted using nonlinear fitting with the program Origin 2016 (OriginLab).

### Electron microscopy.

Grids for cryo-EM were prepared by plasma cleaning UltrAuFoil Holey Gold films (Electron Microscopy Sciences) for 30 s using a Fischione Instruments model 1070 NanoClean. Samples at 0.4 mg ml^−1^ (4 μl) were applied to the grids for 3 s; blotted using a blot force of 2, a blot time of 4 s, a drain time of 0 s, a relative humidity of 100%, and a temperature of 4°C; and plunge frozen into a bath of liquid ethane using an FEI Mark IV Vitrobot. Data were collected on a Thermo Fisher Scientific FEI Titan Krios operating at an acceleration voltage of 300 kV and a Cs (spherical aberration C3) of 2.7 nm. Images were recorded with a Gatan Summit K2 direct electron detector operating in counting mode (Table 1). The microscope was operated using the Leginon software (54).

Frame alignment was performed using the MotionCor2 package and default parameters with exception of a patch 5 (55). Particles were picked using Topaz: bin 4, radius 15, and default pretrained model (resnet16_u64) (56). Contrast transfer function (CTF) values were estimated using CTFFIND4 (57). Particles were extracted from dose-weighted micrographs, normalized, and binned to 64 × 64 with Relion 3.1 (58). Several cycles of 2D classification were carried out with Relion 3.1 to remove featureless particles. The coordinates for the featureful particles were used to extract a second set of particles at 256 × 256 with Relion 3.1. These particles were uploaded to cryoSPARC for further processing (59). *Ab initio* structure determination, heterogeneous classification, 3D variability, nonuniform refinement, and 3D Fourier shell correlation (3DFSC) were performed with cryoSPARC to generate Rep bound to ssDNA and dsDNA (60). 3D variability was employed to separate dsDNA- versus ssDNA-bound particles and separate particles whose *ED* could be visualized.

### Structure determination and analysis.

The coordinates were manually built into the cryo-EM map using Coot (29). The high contents of Arg, Phe, Tyr, and Trp amino acids were particularly helpful in attaining the appropriate amino acid sequence to density register. Structure refinement with the Phenix package (phenix.real_space_refine) was used to improve the geometry (39). An excess of 20 manual building and refinement cycles was necessary to improve the model to sufficient quality for interpretation and deposition. Structure validation was performed using the phenix.validation_cryoem, MolProbity score was determined using phenix.molprobity, and EMRinger score was determined using phenix.emringer (39, 61, 62).

Searches for similar folds of *AD* were performed using the DALI server (31). The subunit-subunit, subunit-ssDNA, subunit-dsDNA, and subunit-ADP interactions were identified using the PDBePISA server and LigPlot+ (63, 64).

Coordinates for the dsDNA cryo-EM map were generated via rigid body refinement of the coordinates derived from the 3.8-Å map. Initial models for 36 nucleotides per strand of A-DNA (twist 32.7°, rise 2.55 Å), B-DNA (twist 36.0°, rise 3.38 Å), C-DNA (twist 40.0°, rise 3.32 Å), and RNA (twist 32.7°, rise 2.55 Å) were generated by the x3DNA server (37). These models were computationally fit into the density using the program MOLREP from the CCP4 package (41). The protein-nucleotide coordinates were further refined using phenix.real_space_refine (39).

Map segmentation was performed using UCSF Chimera with a 2.5-Å-radius color zone. Correlation coefficient values between maps were also calculated using UCSF Chimera (38).

### Identification of nucleic acid bound to Rep.

To determine the identity of nucleic acid bound to Rep, we independently treated the purified sample with DNase I or RNase I (Thermo Fisher) according to the manufacturer’s protocol. For each reaction, a total of 40 μM purified Rep (10 μl) was first denatured at 90°C for 15 min. The sample was cooled to room temperature and then digested with 10 U of RNase I in 20 mM Tris-acetate (pH 8.0), 100 mM NaCl, and 0.1 mM EDTA or with 1 U of DNase I in 100 mM Tris-HCl (pH 7.5), 25 mM MgCl_2_, 1 mM CaCl_2_, and 50 mM EDTA for 2 h. A 1% agarose gel (40 ml) was cast in the presence of 1 μl of SYBR-Gold nucleic acid stain (10,000× concentrate in dimethyl sulfoxide [DMSO]; Thermo Fisher). For controls, a 1 μM concentration of a 12- and a 44-nt oligonucleotide (10 μl each) was also processed with the agarose gel. Gels were visualized using a UV-transilluminator at 302 nm.

### Data availability.

The cryo-EM map and atomic coordinates have been deposited into the EMDB and PDB, respectively. Accession codes (EMDB/PDB) are as follows: Rep with ssDNA, 23249/7LAR, and Rep with dsDNA, 23250/7LAS, respectively.
